# Yeast Biodiversity in Vineyard Environments Is Increased by Human Intervention

**DOI:** 10.1371/journal.pone.0160579

**Published:** 2016-08-08

**Authors:** João Drumonde-Neves, Ricardo Franco-Duarte, Teresa Lima, Dorit Schuller, Célia Pais

**Affiliations:** 1 CITAA - Research Center for Agricultural Technology of Azores, University of Azores, Angra do Heroísmo, Portugal; 2 Center of Molecular and Environmental Biology (CBMA), Department of Biology, University of Minho, Braga, Portugal; Leibniz-Institut fur Pflanzengenetik und Kulturpflanzenforschung Gatersleben, GERMANY

## Abstract

One hundred and five grape samples were collected during two consecutive years from 33 locations on seven oceanic islands of the Azores Archipelago. Grape samples were obtained from vineyards that were either abandoned or under regular cultivation involving common viticultural interventions, to evaluate the impact of regular human intervention on grape yeast biota diversity in vineyards. A total of 3150 yeast isolates were obtained and 23 yeast species were identified. The predominant species were *Hanseniaspora uvarum*, *Pichia terricola*, *Starmerella bacillaris* and *Issatchenkia hanoiensis*. The species *Barnettozyma californica*, *Candida azymoides* and *Pichia cecembensis* were reported in grapes or wine-associated environments for the first time. A higher biodiversity was found in active vineyards where regular human intervention takes place (Shannon index: 1.89 and 1.53 in the first and second years, respectively) when compared to the abandoned ones (Shannon index: 0.76 and 0.31). This finding goes against the assumptions that human intervention can destroy biodiversity and lead to homogeneity in the environment. Biodiversity indices were considerably lower in the year with the heaviest rainfall. This study is the first to report on the grape yeast communities from several abandoned vineyards that have undergone no human intervention.

## Introduction

Yeasts have been used for millennia by humankind in the production of fermented foods. They are considered one of the first organisms to be domesticated and they are closely linked to the history of civilization, cultures, and economies [1,2]. Wine is one of the most important fermented beverages, vineyards and grapes being the primary source of natural yeasts in wine production [3]. Grape juice composition and the microbiota conducting the fermentation are the most important factors affecting wine quality [4]. A countless number of studies reporting on grape yeast diversity show that the distribution and abundance of yeast communities are shaped by both natural and anthropogenic environmental factors. Yeast biodiversity is influenced by climate, vineyard location, grape cultivar, and by ripeness and health of the grape berries [3,5–11]. Human interventions also have an impact on yeast biodiversity. Different farm strategies for pest/disease/weed control and soil maintenance (irrigation, fertilization and soil cover) differently influence grape health and development, having direct or indirect effects on the survival and dispersal of yeasts and their vectors [12–16]. Likewise, canopy management practices such as pruning, training, thinning and leaf removal affect the microclimate at the level of the berries, affecting therefore yeast survival and growth on grape berries [3,17].

Human activities are essential in the shaping of agro-ecosystems, which raises the question of the overall impact of human intervention on grape yeast biodiversity. The Archipelago of the Azores is a suitable model to address this question because islands represent simplified real-world systems and in most of the Azorean islands there are both abandoned vineyards and vineyards that are under regular cultivation (active vineyards). Since the formulation of Charles Darwin’s evolutionary theory in 1859, oceanic islands have been recognized as preferential model systems for research in biogeography, ecology, and conservation, considering that their well-defined borders facilitate the observation of ecological processes [18]. The Azores Archipelago is composed of nine volcanic islands located in the mid-Atlantic Ocean, between 36° 55' and 39° 43' latitude N and 25° 00' and 31° 17' longitude W. Grapevine was introduced in Azores with the settlement of the archipelago in the fifteenth century. Viticulture expanded to most of the islands and was practiced in incipient soils (unchanged solidified lava flows or very stony soils). In the nineteenth century, the cultivars were replaced with American grape cultivars and their hybrids due to fungal grape diseases (powdery mildew and downy mildew) and *Phylloxera* [19]. Today, European cultivars occur predominantly in the appellations of origin of the Pico, Terceira, and Graciosa Islands. Some of these *terroirs* are classified by UNESCO as world heritage “landscape of the Pico Island vineyard culture” (http://whc.unesco.org/en/list/1117). During the last decades, social and economic change caused the abandonment of more than 85% of the archipelago’s total vineyards area. Once abandoned, grapevines can continue to bear fruit for a few more years, competing with the endemic and/or exotic floras as they attempt to reestablish themselves.

The aim of this study was to evaluate the impact of viticultural practices and regular human intervention on the diversity of the grape yeast microbiota. Island environments with extensive areas of abandoned vineyards, with no direct human intervention for years, provide a suitable model. We used culture-dependent methods to compare the yeast community composition of grapes grown in abandoned and active vineyards in the Azores islands.

## Material and Methods

### Sampling and yeast isolation

**Fig 1:**
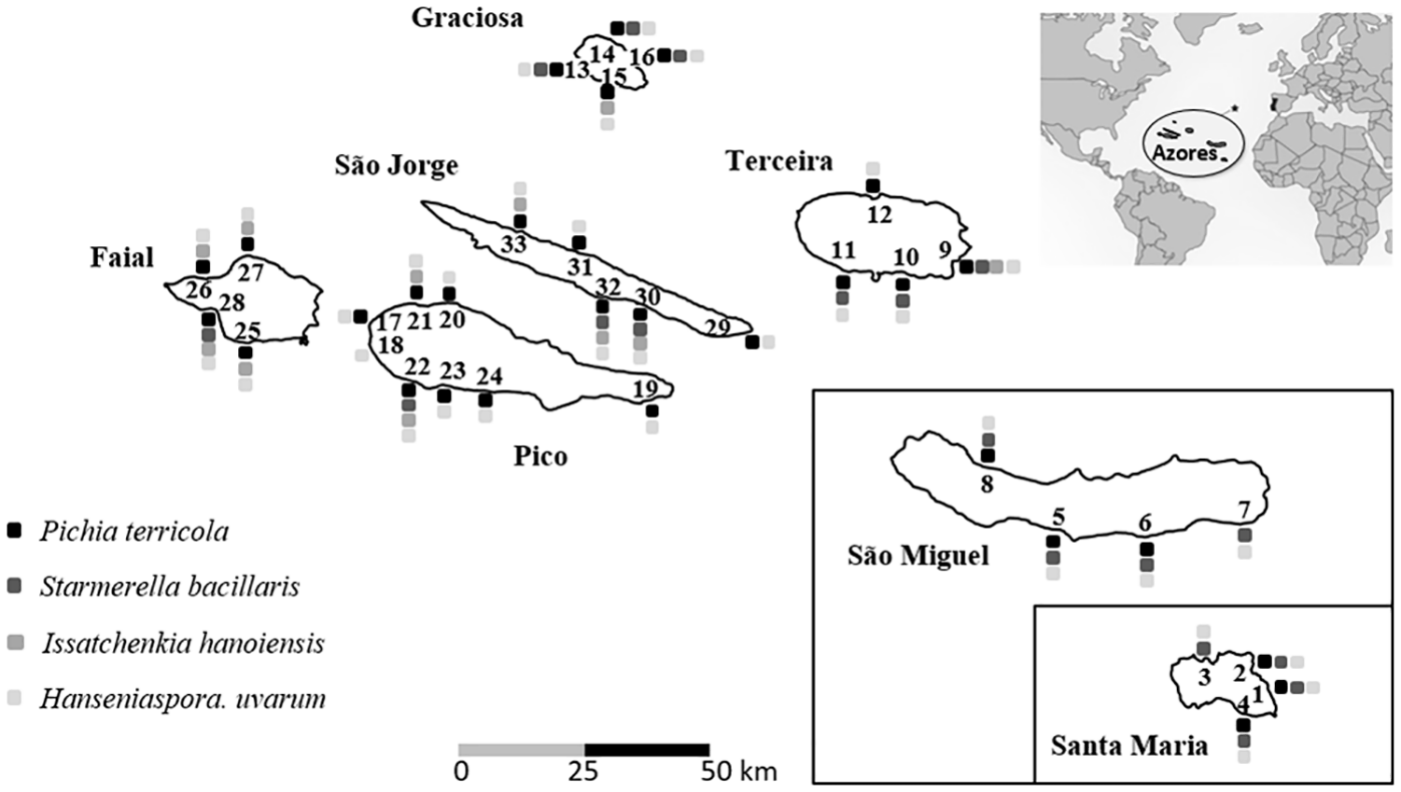
Location of 33 sampling sites on the islands of the Azores Archipelago, indicating the occurrence of the four most abundant yeast species (*S*. *bacillaris*, *H*. *uvarum*, *I*. *hanoiensis* and *P*. *terricola*).

Grape samples were collected from 33 locations on seven islands of the Azores Archipelago during the 2009 and 2010 harvests (57 and 48 grape samples, respectively) (Fig 1 and S1 Table), always with the permission of the land owners. Locations were chosen regarding the existing vineyards, the number of locations per island being independent of its area. Grapes of the species *Vitis labrusca* L. cultivars and its hybrids were collected in vineyards that were either abandoned for at least five years or in constant cultivation through regular viticultural interventions (active). The distance between abandoned and active vineyards on the same location ranged between 300 and 700 m. Each sample consisted of approximately 2–3 kg of rot-free grape bunches that were collected aseptically into sterile plastic bags and immediately transported to the laboratory under refrigerated conditions. For each location grape bunches were harvested in four different sampling points, separated by an average distance of *ca*. 250 m, in order to obtain a high diversity inside each harvest location. The berries were manually crushed inside the sterile bags and diluted aliquots (10^−1^ to 10^−5^) of the grape juice were spread on plates containing YPD medium (yeast extract, 1% w/v; peptone, 1% w/v; glucose 2% w/v and agar 2%, w/v) supplemented with biphenyl (40 mg/L). After incubation (2 days, 30°C), 30 colonies were randomly collected from plates containing between 30 and 300 colonies, which corresponded to a dilution of 10^−2^. A total of 3150 yeast isolates was obtained and stored in glycerol (30%, v/v) at -80°C.

### Molecular identification of the yeast isolates

DNA extraction was performed according to Drumonde-Neves [20]. Molecular identification of isolates was performed by restriction fragment length polymorphism analysis (RFLP) and subsequent DNA sequencing of distinct electrophoretic patterns. The 5.8-S ITS region was amplified using the primers ITS1 (5'-TCCGTAGGTGAACCTGCGG-3') and ITS4 (5'-TCCTCCGCTTATTGATATGC-3') [21]. PCR was performed as follows: initial denaturation at 95°C for 6 min; 35 cycles of denaturing at 95°C for 20 s, annealing at 53°C for 20 s, extension at 72°C for 1 min; final extension at 72°C for 5 min. PCR amplification was carried out in a final volume of 10 *μ*L of a reaction mix containing 20–50 ng of yeast DNA, 0.5 U *Taq* polymerase (MBI Fermentas), 1x *Taq* buffer (10 mM Tris-HCl, 50 mM KCl, 0.08% Nonidet P-40), 0.4 pmol of each primer, 0.2 mM of each deoxynucleotides and 1.5 mM of MgCl_2_. After dilution (1:4), 10 *μ*L of the PCR products were digested with the restriction endonuclease *Hinf*I (Fermentas) according to the supplier’s instructions. PCR products and their restriction fragments were mixed and separated in a 2% (w/v) agarose gel containing GelRed^™^, (1x TAE Buffer, 100 V, 75 min). Identical electrophoretic profiles of each sample were considered as conspecific and grouped and one representative isolate per group was selected for sequencing of the 5.8-S ITS region. The amplicons were obtained as described above and sequenced by the Sanger method [22,23]. Sequence reactions were performed using the forward primer ITS1 and the BigDye Terminator Cycle Sequence Ready Reaction Kit version 3.1 (Applied Biosystems, Foster City, CA). After the sequence reaction, excess dye terminators were removed by gel filtration. Sequences were analyzed with an automated DNA sequencer 3730XL (Applied Biosystems). Species identity was determined using the BLASTN program [24] and GenBank reference sequences, considering an identity threshold of at least 98%.

### Statistical analysis

The ecological evaluation was based on species richness (S), which represents the total number of species found in a defined area (community), the Shannon index (H), which assesses the general biodiversity, accounting for both the abundance and evenness of the species present [25], and the equitability index (E_H_), which evaluates how equal the community is numerically and refers to how similar in numbers are the species in a grape sample microenvironment [26]. The null hypothesis of no difference in yeast communities composition and biodiversity between vineyards in different cultivation status (abandoned and active), between islands, and between sampling years was tested by the Student’s t-test.

## Results

### Yeast species occurring on vineyards of the Azores Archipelago

A total of 3150 yeast isolates were obtained from vineyards of seven islands of the Azores archipelago. As shown in Table 1, 23 yeast species representing 11 genera were found. A total of 1920 isolates were found in active vineyards, whereas for abandoned vineyards 1230 isolates were recovered. Considering the distribution per year, a total of 1710 and 1440 isolates were obtained in 2009 and 2010, respectively. The predominant species was *Hanseniaspora uvarum*, corresponding to 66% of the isolates. *Pichia terricola*, *Starmerella bacillaris*, and *Issatchenkia hanoiensis*, representing 10.9%, 7.67% and 2.51% of the isolates, respectively, were also predominant.

**Table 1:** Number and distribution of species collected on the islands of Azores.

	Island																												Percentage among total number of isolates
	Santa Maria				São Miguel				Terceira				Graciosa				Pico				Faial				São Jorge				
	2009		2010		2009		2010		2009		2010		2009		2010		2009		2010		2009		2010		2009		2010		
	AC	AB	AC	AB	AC	AB	AC	AB	AC	AB	AC	AB	AC	AB	AC	AB	AC	AB	AC	AB	AC	AB	AC	AB	AC	AB	AC	AB	
Species / N° of isolates	120	120	90	120	120	0	120	0	120	90	90	60	120	120	120	0	240	210	240	180	120	60	120	30	150	120	150	120	
*Barnettozyma californica*					2																								0.06
*Candida azyma*		8																											0.25
*Candida azymoides*																		30											0.95
*Candida carpophila*														3											30				1.05
*Candida diversa*	1				1												46	4											1.65
*Candida incommunis*																					1								0.03
*Candida quercitrusa*		2																											0.06
*Candida railenensis*	11	9									1																		0.64
*Hanseniaspora uvarum*	56	84	86	117	29		103		25	1	83	47	95	91	98		108	138	196	173	45	31	104	30	75	54	91	111	65.9
*Hanseniaspora vineae*																	2												0.06
*Issatchenkia hanoiensis*					6					1							1	11	2		17	15	2			9	15		2.51
*Metschnikowia pulcherrima*															1		22	11											1.08
*Meyerozyma guilliermondii*																	6												0.19
*Pichia cecembensis*												4					6		22										1.02
*Pichia fermentans*																	8												0.25
*Pichia kudriavzevii*																			6										0.19
*Pichia membranifaciens*							2				2	4					3			1					13	1			0.82
*Pichia terricola*	2		4	3	1		11		30	51	2	4	11	10	18		9	8	13	5	41	14	10		32	35	24	6	10.9
*Saccharomycopsis crataegensis*												1							1	1							8		0.35
*Saccharomycopsis vini*							4		19	7	2				3						1		4				11	3	1.72
*Saturnispora zaruensis*																											1		0.03
*Starmerella bacillaris*	44	17			71				25	11			14	16				7			15					21			7.67
*Zygoascus meyerae*					10				20	19							29												2.48

(AC: Active vineyards; AB: abandoned vineyards)

Some trends of preferential occurrence were evident for some species, as opposed to a clearly delimited zonation between islands or groups of islands, as summarized in Fig 1. *S*. *bacillaris* was less frequent in the three islands with higher geographic proximity (Pico, Faial and S. Jorge), occurring only in 4 of 17 locations. This species was present in all locations of the eastern islands (S. Maria and S. Miguel). In contrast, *I*. *hanoiensis* did not occur on the eastern islands, but was found in 11 of the 25 remaining locations. No island-specific preferential occurrence was observed for the most frequent species *H*. *uvarum*, which was present in all 33 locations, and *P*. *terricola* present in 29 out of 33 locations.

**Fig 2:**
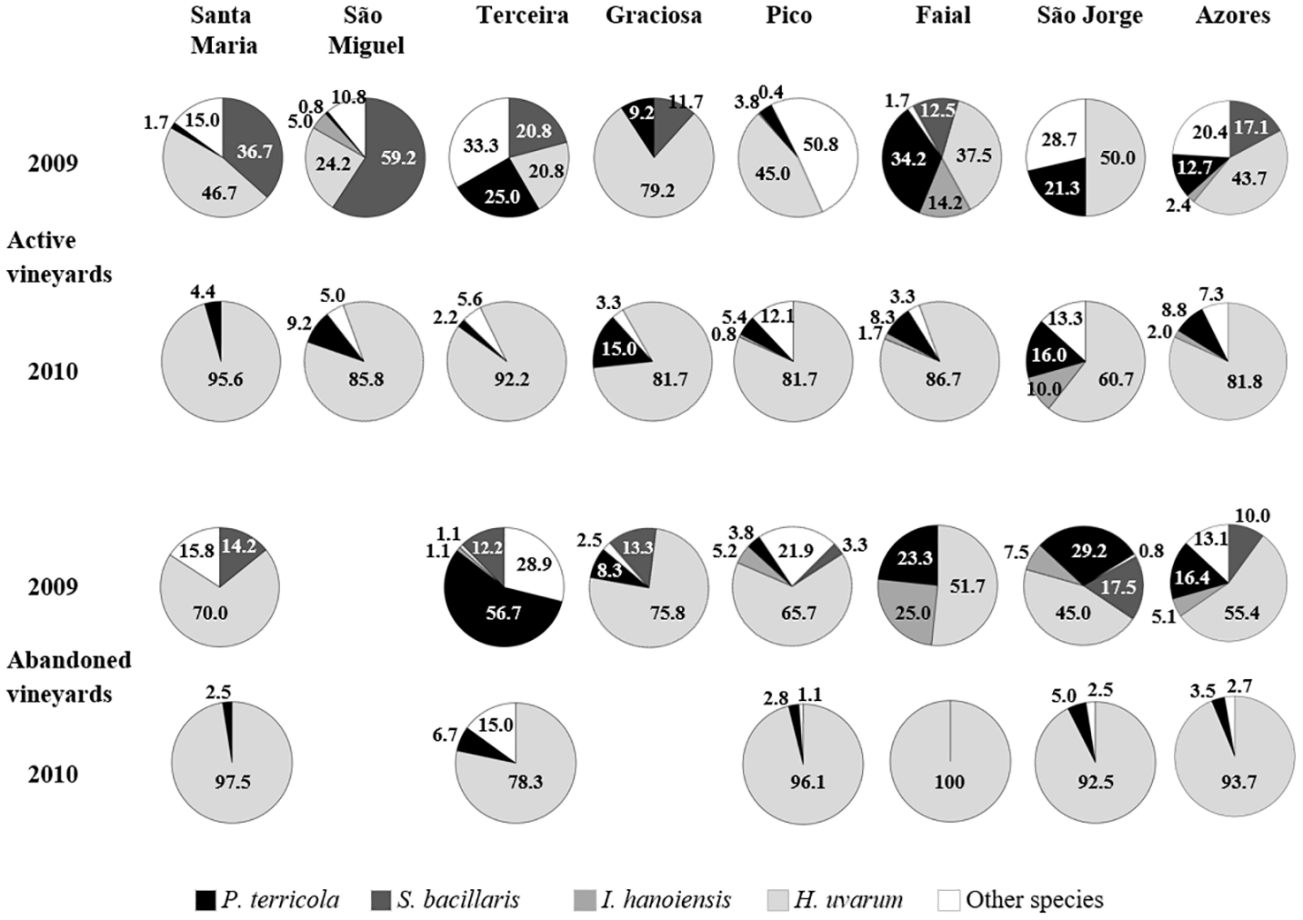
Incidence values in percentage of the four most abundant species on each island and global distribution on the Azores, according to the sampling year in both active and abandoned vineyards in 2009 and 2010.

A detailed comparison of the four predominating species distribution on each island is summarized in Fig 2, as a function of the sampling year in both cultivated and abandoned vineyards. Overall, the yeast biota suffered a strong reduction in species richness from 2009 to 2010 in both types of vineyards. Climatologically, the year of 2009 was characterized by the average conditions expected for the Archipelago, whereas unusually high frequencies of precipitation were recorded in 2010. Rainfall on the central and eastern islands was 20% and 60% higher, respectively, compared to the average values of the previous 30 years. The predominant species *H*. *uvarum* contributed to the total yeast biota on the Azorean islands with 44–55% and 82–94% in samples collected in 2009 and 2010, respectively. This species accounted for more than 50% of the isolates on most of the islands with a few exceptions for some active vineyards of sampling year 2009. The species *P*. *terricola*, *S*. *bacillaris*, and *I*. *hanoiensis* contributed to a lower percentage (between 2 and 20%) of the overall Azorean yeast community, whereas their occurrence on each island tended to be heterogeneous and depended also on the vineyard type besides the sampling year. On the islands of S. Maria, S. Miguel, and Terceira, the species *S*. *bacillaris* ranged in 2009 from 21 to 59% in active vineyards and remained less than 15% in abandoned vineyards. *P*. *terricola* was more predominant among the yeast community of Terceira, Faial, and S. Jorge. As already described for the predominating species, the contribution of other yeast species (detailed in Table 1) was higher in 2009 (20.4 and 13.1 for active and abandoned vineyards, respectively) compared to 2010 (7.3 and 2.7 for active and abandoned vineyards, respectively).

### Yeast biodiversity varies according to sampling years and cultivation modes

**Fig 3:**
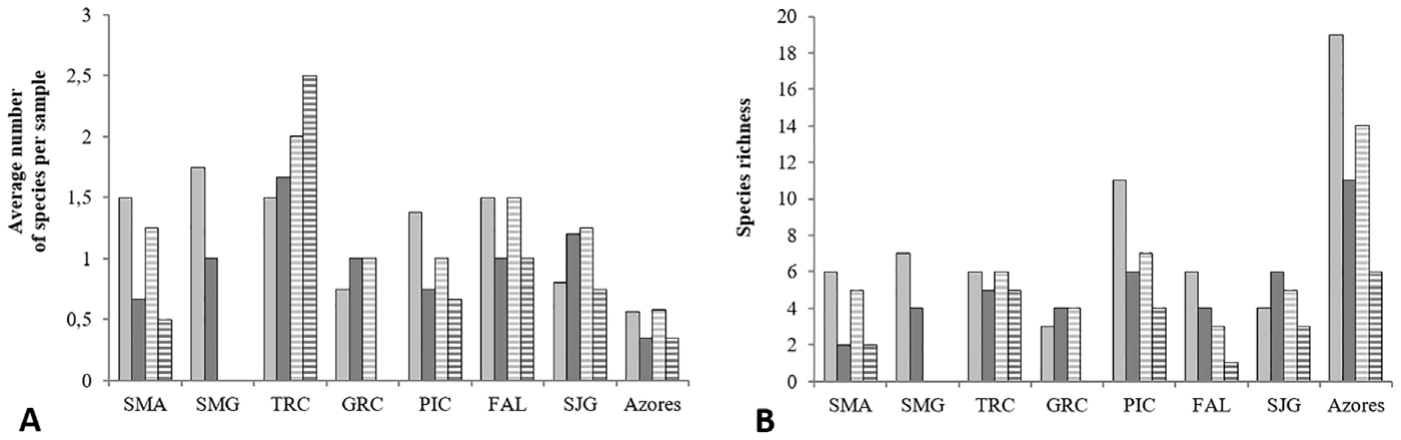
Average number of species per sample (A) and species richness (B) for each island (SMA: Santa Maria, SMG: São Miguel, TRC: Terceira, PIC: Pico, FAL: Faial, SJG: São Jorge) and for the global Azorean yeast communities from all islands, in active and abandoned vineyards and two consecutive sampling years (light grey squares- 2009, active vineyards; dark grey squares- 2010, active vineyards; light grey striped squares- 2009, abandoned vineyards; dark grey striped squares- 2010, abandoned vineyards).

The species richness, the average number of species per sample and the biodiversity indices (Shannon and equitability) were compared for each island and for the overall Azorean yeast biota of all islands according to the sampling year in both active and abandoned vineyards (Fig 3 and Table 2).

**Table 2:** Biodiversity indices (Shannon and equitability) for each island (SMA: Santa Maria, SMG: São Miguel, TRC: Terceira, PIC: Pico, FAL: Faial, SJG: São Jorge) and for the global Azorean yeast communities from all islands, in active (AC) and abandoned (AB) vineyards and two consecutive sampling years.

*Shannon index*	SMA	SMG	TRC	GRC	PIC	FAL	SJG	Azores
2009_AC	1.2	1.16	1.63	0.65	1.69	1.35	1.21	1.89
2010_AC	0.18	0.53	0.38	0.58	0.7	0.51	1.21	0.76
2009_AB	0.97		1.21	0.78	1.18	1.03	1.26	1.53
2010_AB	0.12		0.8		0.2	0	0.31	0.31
*Equitability index*								
2009_AC	0.67	0.6	0.91	0.6	0.7	0.75	0.87	0.64
2010_AC	0.26	0.38	0.24	0.42	0.39	0.37	0.67	0.32
2009_AB	0.6		0.67	0.56	0.61	0.94	0.78	0.58
2010_AB	0.17		0.5		0.14	0	0.29	0.18

The number of grape samples collected on most islands was dependent on the areas of active and abandoned vineyards. For example, from Pico Island (S2 Table), where the vineyards area is the largest, twice as many samples were collected, than from the other islands. In 2010, due to bad weather conditions, it was not possible to obtain samples from all locations. The average number of species per sample (Fig 3A) in each island varied between 0.5 and 2.5, being highest in Terceira Island, showing no correlation with a higher or lower number of samples collected. Fig 3B shows the total number of species found per island, sampling year, or cultivation scheme. In agreement with the data presented in the previous section, species richness on the Azores was higher in 2009 (total 14–19 species, ranging from 3 to 11 per island) than in 2010 (total 6–11 species, ranging from 1 to 6 per island). In active vineyards we found in both sampling years a higher species richness (11–19 species, ranging from 2 to 11 per island) compared to the vineyards that were abandoned for at least five years (total 6–14 species, ranging from 1 to 7 per island). This pattern was apparent on most islands, and clearly evident on Pico Island, where the highest number of samples was collected.

To compare the biodiversity of all islands as a function of sampling year and cultivation mode, the Shannon and equitability indices were determined as measures of species diversity and evenness within a community (Table 2). The overall Shannon indices for yeast species on the Azorean islands in 2009 were 1.9 for active vineyards and 1.5 for abandoned vineyards. The corresponding values in 2010 were 0.8 and 0.3, respectively. The same tendency was observed for the equitability index. On almost all islands and for both sampling years, the biodiversity measures were always higher in samples collected from cultivated vineyards. For these samples, the highest values in 2009 were obtained, followed by the same sampling sites in 2010. The lowest overall values for biodiversity parameters were achieved in non-cultivated locations in 2010. Minor exceptions were only found for Graciosa and Terceira Islands, for which the lowest values were obtained in active vineyards in 2010. Our results are strongly supported by the repeated observation of a lower biodiversity in abandoned vineyards in two consecutive sampling years that were very different in terms of climate, and also by the fact that observations were made on independent islands, two of them (S. Miguel and S. Maria) being located at a distance of about 200 km from the central group of islands.

Statistical comparisons were made regarding the yeast community composition between islands, sampling years, and cultivation schemes. The number of isolates per sample of the most representative species (*S*. *bacillaris*, *H*. *uvarum*, *I*. *hanoiensis* and *P*. *terricola*), and the Shannon and equitability indices were calculated for each of the 105 individual grape samples and compared by Student’s t-test as a function of sampling years and cultivation status. Significant differences among islands were found for the incidence values of the species and also for the biodiversity indices. Table 3 shows a pairwise comparison of yeast biota isolates in 2009 and 2010, in active and abandoned vineyards. In the sampling year 2009, 19 statistically significant differences were found when the distribution of the four species in active vineyards of all islands was compared, whereas only seven such differences were apparent for abandoned vineyards. One and two significant differences were found for the Shannon and equitability indices in cultivated and abandoned vineyards in the previously mentioned sampling years, respectively. These data suggest the presence, in vineyards that are cultivated, of a more divergent yeast microbiota that tends to become more homogeneous in abandoned vineyards. This is even more evident for 2010, when no significant differences were observed between islands in the yeast microbiota from abandoned vineyards. Table 4 shows the same pairwise comparison, but this time comparing the yeast biota obtained in each island in both sampling years. Results show an even higher number of statistically significant differences when the different sampling years were compared, for both active and abandoned vineyards. Overall, there were 51 statistically significant differences for species and islands between both sampling years on active vineyards. By contrast, the number of significant differences was 14 for the comparisons between the two sampling years when abandoned vineyards were considered. The corresponding differences regarding both the Shannon and equitability indices were 10 and 12 in cultivated and abandoned vineyards, respectively. As expected, the majority of statistically significant differences were evident for the incidence of the most frequent species *H*. *uvarum*.

**Table 3:** Statistical analysis between means of predominant species incidences (*Hu*: H. uvarum, *Sb*: S. bacillaris, *Ih*: I. hanoiensis, *Pt*: P. terricola) and between means of biodiversity indices (H: Shannon index, Eh: equitability index) on each island (SMA: Santa Maria, SMG: São Miguel, TRC: Terceira, PIC: Pico, FAL: Faial, SJG: São Jorge), for comparison of yeast biota isolated in 2009 and 2010 in active (AC) and abandoned (AB) vineyards. Number of stars correspond to *p* values equal to 0.05 (*), 0.01 (**) and 0.001 (***).

			*Hu*			*Sb*		*Ih*		*Pt*		*H*	*Eh*	
			*GRC*	*TRC*	*SJG*	*PIC*	*SJG*	*FAL*	*SJG*	*FAL*	*SJG*	*SJG*	*PIC*	*SJG*
*2009*	*AC*	*SMA*				*	*	*		*	**			
		*SMG*	*			*	*			*	**			
		*TRC*	*			*	*	*						
		*GRC*						*						
		*PIC*						*		*	*			
		*FAL*							*			*		
	*AB*	*SMA*		*							***			
		*GRC*		**										
		*PIC*		**							**			*
		*FAL*		**									*	
		*SJG*		*										
*2010*	*AC*	*SMA*			**							**		**
		*SMG*			*							*		
		*TRC*										**		***
		*GRC*										*		
		*PIC*										**		***
		*FAL*			*									*

**Table 4:** Statistical analysis between means of predominant species incidences (*Hu*: H. uvarum, *Sb*: S. bacillaris, *Ih*: I. hanoiensis, *Pt*: P. terricola) and between means of biodiversity indices (H: Shannon index, Eh: equitability index) on each island (SMA: Santa Maria, SMG: São Miguel, TRC: Terceira, PIC: Pico, FAL: Faial, SJG: São Jorge), for comparison of yeast biota between sampling years. Number of stars correspond to *p* values equal to 0.05 (*), 0.01 (**) and 0.001 (***).

			*2009*																				
			*Hu*						*Sb*			*Ih*	*Pt*	*Pt*	*H*					*Eh*			
			*SMA*	*SMG*	*TRC*	*PIC*	*FAL*	*SJG*	*SMG*	*SMA*	*GRC*	*FAL*	*SMA*	*SJG*	*SMA*	*TRC*	*PIC*	*SJG*	*FAL*	*TRC*	*SMA*	*FAL*	*SJG*
*2010*	*AC*	*SMA*	**	**	**	**	*	*	*	*		*	*		*								
		*SMG*		**	**	*		*	*	*		*	*										
		*TRC*		*	*	*				*		*	*										
		*GCR*		**	**	*			*	*		*	*					*					
		*PIC*		**	**	*			*	*	*	*	*		*					*			
		*FAL*		**	**	*	*	*	*	*					**	*	*			*	*		
		*SJG*		*	*				*	*			*					*					
	*AB*	*SMA*			***		**	*						**				**	**			*	***
		*TRC*			*									*									
		*PIC*			***		***	*						***				***	***			**	***
		*SJG*			***		**	*						**				*	*			*	**

## Discussion

This study aimed to investigate the consequences and extent of regular human intervention in the biodiversity of grape yeasts. A two-year sampling plan was designed and implemented at 33 locations in seven islands of the Azores Archipelago. A detailed description of the yeast microbiota in those vineyards was presented, which is not often done for such remote environments. A total of 105 grape samples was obtained from *V*. *labrusca* L. cultivars, which are rarely studied, in abandoned and active vineyards. 3150 yeast isolates were analyzed and 23 yeast species belonging to 11 genera were identified. We must consider that yeast isolates were obtained through selective growth conditions that may differ from abiotic factors found in nature. Rarely occurring or slow-growing species may not have been detected, as the detection limit of our experimental approach is 3.3% (one species in 30 isolates). Also, when sequencing one representative per grape sample (in a total of 450 profiles sequenced), some diversity could be lost in the remaining ones. However, we consider that our approach allows the comparison of the yeast microbiota across vineyards and islands, even though it cannot provide a complete description of yeast community composition. We should also point out that although abandoned vineyards were not subject to pest control practices, only ripe and undamaged grapes were collected.

Independently of sampling year, our results show that the yeast community of active vineyards was more divergent between islands and harbored a higher biodiversity compared with the microbiota of abandoned vineyards. This may result from the impact of human intervention on those agro-ecosystems. Regular management practices performed in vineyards under cultivation, as for example the effect of dissemination by agricultural machinery or the effect of the workers themselves, may provide a wide range of opportunities for yeast colonization, survival, and growth, which may explain the higher biodiversity occurring in these vineyards. Overall, on each island and sampling location, abandoned and active vineyards shared similar geographical, climatic and soil constraints. The absence of agricultural practices in abandoned vineyards led to a lower yeast biodiversity in those ecosystems but also to a certain homogeneity between islands. This is an important finding that goes against the assumptions that human intervention can destroy biodiversity and lead to homogeneity in the environment.

The most representative yeast genera occurring on grapes from vineyards of the Azores Archipelago are comparable to those identified in other reports dealing with grape microbial communities in continental Europe [27–30], Africa [31], Asia [29,32–34] and South America [35–37]. However, to our knowledge, the species *B*. *californica*, *C*. *azymoides* and *P*. *cecembensis* have not previously been reported in grape or wine associated environments.

The predominant species were *H*. *uvarum*, *P*. *terricola*, *S*. *bacillaris*, and *I*. *hanoiensis*, representing 66%, 10.9%, 7.7% and 2.5% of the total number of isolates, respectively. This is in agreement with the large majority of studies reviewed by [3] and with more recent ones. It is widely reported that the apiculate yeast *H*. *uvarum* (anamorph *Kloeckera apiculata*) is the predominant species on grape berries, freshly crushed grape berries or initial stages of spontaneous fermentations, together with oxidative species of the genera *Candida* and *Pichia* [27,28,33,34,38–42]. The cited studies refer to fruits samples from *Vitis vinifera* cultivars. The few studies that have assessed the yeast microbiota of grapes of *V*. *labrusca* cultivars encountered similar results with respect to the predominant yeast genera and species, in particular *H*. *uvarum* [36,43]. However, Parish and Carroll [44], assessing the yeast community associated with *Vitis rotundifolia* grapes, noted that the absence of *H*. *uvarum* was unusual in comparison with *V*. *vinifera* berries. Another recent report on the yeast microbiota of hybrid cultivar grapes (a cross between *V*. *vinifera* and *Vitis riparia* x *Vitis Rupestris*) found *H*. *uvarum* in low proportions and no members of the genera *Pichia* and *Candida* [45]. Although studies of grapevine species other than *V*. *vinifera* are very few, the yeast community of grapes of those species seems to be similar to that found on fruits of *V*. *vinifera*. When differentiated communities were identified, the underlying cause did not seem to be grapevine species, but instead some other factor such as climatic conditions. Moreover, the effect of the particular yeast isolation methodologies used by Parish & Carroll [44] and Lederer et al. [45] cannot be neglected.

In the present study the distribution of predominant yeast species followed geographic trends. This is in agreement with the view that geographical location influences grape yeast communities [6]. A higher incidence of *S*. *bacillaris* and the absence of *I*. *hanoiensis* in S. Maria and S. Miguel (eastern group) differentiated the grape yeast community of these islands from those of the remaining islands of the archipelago. However, our observations also demonstrate a strong temporal component, showing that ecologically meaningful generalizations require repeated sampling.

Climatologically, 2009 was characterized by the average conditions expected for the Archipelago, whereas unusually high frequencies of precipitation were recorded in 2010. Rainfalls in the central and eastern islands were 20% and 60% higher, respectively, compared to the average values of the last 30 years. In 2010, yeast biodiversity in the vineyards of the archipelago was considerably lower than in the previous year. Overall, a pronounced decrease was observed in species richness (from 34 to 31 and from 24 to 17 in active and abandoned vineyards, respectively), in Shannon’s index (from 1.89 to 0.76 and from 1.53 to 0.31 in active and abandoned vineyards, respectively), and the equitability index (from 0.64 to 0.32 and from 0.58 to 0.18 in active and abandoned vineyards, respectively). It is commonly accepted that climatic and microclimatic conditions affect yeast community composition and dynamics. However, several studies have reached contradictory conclusions. Some studies report higher yeast counts [12] or higher yeast biodiversity [5,46] in rainy and/or cold vintages, but our results are in agreement with other reports that found lower yeast diversity in rainy years [47,48]. This fact has been attributed to the effect of antifungal treatments that are carried out with a greater intensity in more humid years [44]. In a comparison of the yeast microbiota of one abandoned vineyard with that of two groups of vineyards where fungicides were applied, Guerra et al. [49] concluded that pesticide application might affect yeast diversity. Some studies suggest that the use of pesticides in vineyards decreases yeast biodiversity [50] and others conclude that the presence of several fungicides has a minor impact on the composition of grape berry communities [12]. In organic vineyards, where the use of fungicides is limited to the application of sulphur and copper sulphate, the yeast biodiversity can be lower than in vineyards under conventional management [15,51]. Certain pesticides may even stimulate yeast species such as *K*. *apiculata* (*H*. *uvarum*) [52]. The lower biodiversity observed in 2010 was evidently the consequence of the more intense rainfall itself and not the effect of pesticides, given that abandoned vineyards were not treated. This was also associated with a strong predominance of *H*. *uvarum* in 2010 (overall increase was 38%). As reviewed by Fleet et al. [3] some mechanisms of entrapment, adsorption, or attachment of yeast cells to the grape exocarp prevent them from being washed away by rainfall. A similar mechanism may be involved in higher incidence of *H*. *uvarum*, compared to other species, in rainy years. This is in agreement with the fact that this species is obtained in considerably higher proportions when isolated from crushed grapes instead of the surface of grape skins using washing solutions [16,53,54].

To the best of our knowledge, this study is the first report on the yeast community of grapes growing in several abandoned vineyards, free from human intervention. It is also the first in which yeast diversity from grapes grown in remote oceanic islands is assessed.

## Supporting Information

S1 Table Number and distribution of collected samples in the 33 locations of the 7 Azorean islands. (XLSX)

S2 Table Number and distribution of species collected in the islands of Azores, in two sampling years (2009 and 2010) and in active (AC) and abandoned (AB) vineyards. (XLSX)
